# A Randomized Controlled Trial on the Effects of Electromyographic Biofeedback on Quality of Life and Bowel Symptoms in Elderly Women With Dyssynergic Defecation

**DOI:** 10.3390/ijerph16183247

**Published:** 2019-09-04

**Authors:** Miguel A. Simón, Ana M. Bueno, Patricia Otero, Fernando L. Vázquez, Vanessa Blanco

**Affiliations:** 1 Health Psychology Research Unit, Department of Psychology, University of A Coruña, 15071 A Coruña, Spain (A.M.B.) (P.O.); 2 Department of Clinical Psychology and Psychobiology, University of Santiago de Compostela, 15782 Santiago de Compostela, Spain; 3 Department of Evolutionary and Educational Psychology, University of Santiago de Compostela, 15782 Santiago de Compostela, Spain

**Keywords:** behavioral treatment, chronic constipation, dyssynergic defecation, EMG-biofeedback

## Abstract

Dyssynergic defecation is a usual cause of chronic constipation in elderly women, with a negative impact on health-related quality of life. The present randomized controlled trial aims to evaluate the effects of behavioral treatment through electromyographic biofeedback (EMG-BF) on quality of life and bowel symptoms in elderly women with dyssynergic defecation. Twenty chronically constipated elderly women, due to dyssynergic defecation, were enrolled in the study. Outcome measures included weekly stool frequency, anismus index, severity of patient-reported chronic constipation symptoms (abdominal, rectal, and stool symptoms), and overall measure of quality of life. After 1 month of baseline, participants were randomly assigned to either EMG-BF group (*n* = 10) or control group (*n* = 10). Three months after treatment, female patients were once again assessed following the same procedure in baseline. One-way multivariate analysis of variance MANOVA revealed no significant differences between the groups before treatment in any of the measured dependent variables (Wilks’s λ = 0.74; F_6,13_ = 0.77; *p* = 0.61). Likewise, univariate analysis showed no differences between the groups, either in terms of age (F_1,18_ = 0.96; *p* = 0.34) or mean disease duration (F_1,18_ = 2.99; *p* = 0.11). Three months after treatment, MANOVA revealed statistically significant differences between the groups (Wilks’s λ = 0.29; F_6,13_ = 5.19; *p* < 0.01). These differences were significant in all outcome measures. EMG-BF produces significant improvements in bowel symptoms and health-related quality of life of elderly women with dyssynergic defecation.

## 1. Introduction

Chronic constipation is the most frequent gastrointestinal disorder in the clinical practice with older people. Its prevalence increases with age (particularly after 60–65 years) [1,2], is significantly more common in women than in men, with rates two to three times higher [3,4], and can significantly affect the health-related quality of life in patients with more severe symptoms [5].

Dyssynergic defecation is highly prevalent in chronic constipation [6,7]. This disorder is characterized by failure to complete defecation due to either paradoxical contraction of pelvic floor muscles or failure to relax these muscles, singularly the puborectalis muscle and the external anal sphincter, to enable the expulsion of stool from the rectum [8,9].

Several controlled trials have found that biofeedback therapy, particularly electromyographic biofeedback (EMG-BF), is the most effective treatment for dyssynergic defecation patients [10,11,12,13,14]. Moreover, the efficacy of biofeedback therapy is maintained during long follow-up periods (more than 2 years) [15,16,17] and no adverse effects have been found derived from its use. For these reasons, the task force of The American Neurogastroenterology and Motility Society and the European Society of Neurogastroenterology and Motility specifically recommend the biofeedback therapy for the treatment of dyssynergic defecation [18]. However, more research is needed to determine the clinical efficacy of biofeedback therapy for the management of dyssynergic defecation in chronically constipated elderly patients, particularly in women, because so far only a single controlled trial has been conducted in this collective [19].

The main purpose of biofeedback is to restore a normal defecation pattern, teaching patients to defecate by relaxing the pelvic floor muscles, specifically the external anal sphincter, while coordinately applying an abdominal adequate propulsive force toward the rectum and anal canal [20]. To achieve this objective, biofeedback techniques provide visual and/or auditory feedback obtained from the anal or intra-anal electromyography or using anorectal manometry [21], although the first one (EMG-BF) has been the most widely used [22].

Recent studies conducted in elderly people with dyssynergic defecation have shown that these patients, besides presenting an increase in the EMG-activity of the external anal sphincter (EAS) during defecation attempts, manifest more pain during defecation and more difficulties to defecate than patients with chronic constipation but without dyssynergic defecation [23]. This result suggests the clinical value of rectal and stool symptoms to differentiate dyssynergic defecation patients from those with other subtypes of chronic constipation and, for this reason, could be used as a relevant measure in outcome research.

Moreover, patients with chronic constipation due to dyssynergic defecation show impaired health-related quality of life [24]. However, despite this finding, at the moment there are no studies aimed at evaluating the possible impact of biofeedback therapy on the improvement of quality of life in elderly women with dyssynergic defecation. This point is very important, because women have more severe constipation symptoms than men and differ in several physiologic parameters at an anorectal level [25]. In addition, this study is potentially very useful because some physicians and patients may question the utility of biofeedback therapy in elderly patients.

The aim of this randomized controlled study is to evaluate the effects of behavioral treatment through EMG-BF on quality of life and abdominal, rectal, and stool symptoms in elderly women with dyssynergic defecation.

## 2. Materials and Methods 

### 2.1. Sample

Fifty-one elderly women without cognitive impairment and with chronic constipation unresponsive to routine management of constipation were selected for the study among the users of a day care center for older people. Three females declined to participate, and twenty-eight did not meet the Rome III criteria for the diagnosis of dyssynergic defecation [26]. The twenty elderly women who fulfilled the inclusion criteria of Rome III were enrolled in the study, during which they did not receive any other treatment or intervention (i.e., they did not use any medication or laxatives or receive any enemas). The age of participants ranged from 67 to 83 years (mean: 75.2), and the mean disease duration was 9.3 years (range: 7 to 14). All subjects gave written informed consent, conducting the research protocol with the approval of the local ethics committee (code number: 1417/2; 14 November 2016) and in accordance with the Declaration of Helsinki.

### 2.2. Outcome Measures

Outcome measures included weekly stool frequency, anismus index, severity of patient-reported chronic constipation symptoms (abdominal, rectal, and stool symptoms), and overall quality of life measure. To record the frequency of bowel movements, a stool diary was provided to the patients and employed throughout the study. Anismus index was calculated as the quotient between EMG-activity (µV) of the external anal sphincter during straining to defecate and EMG-activity (µV) of the external anal sphincter during squeezing. EMG-activity was recorded using an intra-anal plug electrode (12 mm diameter, 45 mm total length). Finally, to assess the severity of patient-reported chronic constipation symptoms and quality of life, two self-report questionnaires were employed: the Patient Assessment of Constipation-Symptoms (PAC-SYM) [27] and the Patient Assessment of Constipation-Quality of Life (PAC-QOL) [28]. The PAC-SYM is a 12-item instrument developed for assessing the severity of patient-reported chronic constipation symptoms. The questionnaire is divided into three symptom subscales (abdominal, rectal, and stool), and the various items are scored on a 5-point Likert scale ranging from 0 (absence of symptoms) to 4 (very severe). So, lower scores indicate lesser severity of symptoms in each of those domains and can be used in a reliable and valid form to detect clinical changes over time and, in this way, to evaluate the response to treatment of chronic constipation [29]. For its part, the PAC-QOL is a 28-item questionnaire that assesses the influence that constipation has on daily life. This instrument allows us to obtain an overall quality of life measure that varies from 0 to 4, where the lowest scores are indicative of a lesser impact of constipation on the person’s quality of life. The PAC-QOL has proven to be internally consistent, valid, and responsive to improvements over time in the burden of chronic constipation on the patient’s daily functioning [28].

### 2.3. Procedure

During a 1-month baseline period, patients recorded their bowel movements in the stool diary and participated in a psychophysiological record session (duration of 45 minutes approximately) to record the EMG-activity of the external anal sphincter in two conditions: during straining to defecate (simulated defecation) and during squeezing (voluntary contraction). Subjects also answered the PAC-SYM and the PAC-QOL questionnaires.

After the baseline period, participants were randomly allocated to the study groups by an independent professional using a random number table: EMG-BF group (*n* = 10) and control group (*n* = 10). Patients of the biofeedback group received eight treatment sessions (two per week) across 1 month. Guided by the therapist and by the visual and auditory feedback of the EMG-activity of the external anal sphincter during simulated defecation, the patients learned gradually to eliminate the inadequate sphincter contraction. Patients of the control group received eight counselling sessions across 1 month (two per week) focused on an explanation about the bowel functioning and the behavioral and psychophysiological mechanisms involved in the defecation process.

**Figure 1:**
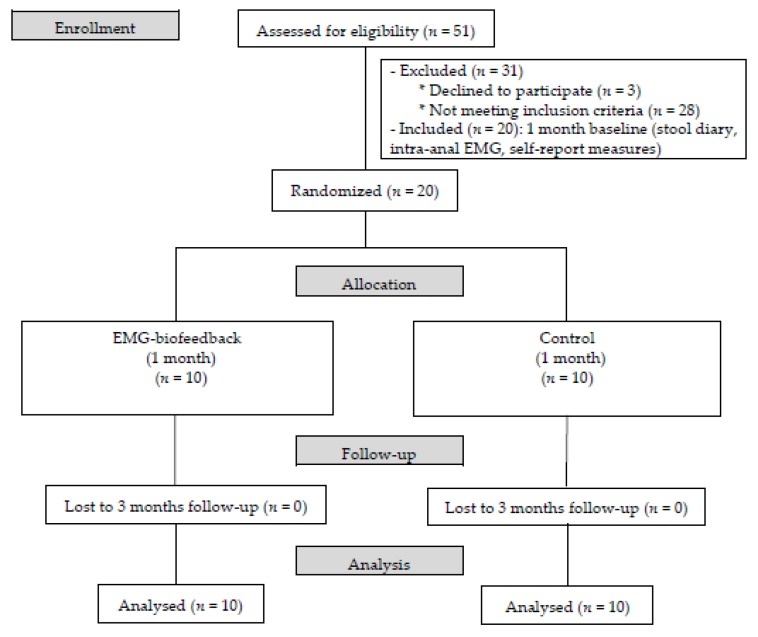
CONSORT flow diagram.

Three months after finishing the intervention, patients of both groups were assessed following the same procedure as in the baseline (they recorded stool diary during 1 month, participated in a psychophysiological record session, and answered the PAC-SYM and the PAC-QOL questionnaires). Figure 1 shows the study flow diagram (CONSORT 2010 flow diagram) [30].

### 2.4. Design

A pre-test–and post-test control group design was employed. Subjects were assigned randomly to groups after completion of the pre-test (baseline). Post-test assessment was done 3 months after finishing the treatment.

### 2.5. Data Analysis

Data analysis was conducted using IBM SPSS Statistics 20 (IBM Corp., Armonk, NY, USA). A MANOVA was performed, with the weekly stool frequency, anismus index, severity of patient-reported chronic constipation symptoms (abdominal, rectal, and stool), and overall quality of life measure serving as the dependent measures. Since this test assumes multivariate normality, this assumption was tested with Box’s M test.

## 3. Results

Anismus index, weekly stool frequency, clinical symptoms (abdominal, rectal, and stool), and overall PAC-QOL scale before and after treatment in both groups are summarized in Table 1. MANOVA revealed no significant differences between the groups before treatment in any of the measured dependent variables (Wilks’s λ = 0.74; F_6,13_ = 0.77; *p* = 0.61) (see Table 2). Previously, the homogeneity of variances was evidenced by Box’s M test (Box M = 13.84; *p* = 0.98). Likewise, univariate analysis showed no differences between the groups either in terms of age (F_1,18_ = 0.96; *p* = 0.34) or in terms of mean disease duration (F_1,18_ = 2.99; *p* = 0.11). The mean EMG-activity (µV) of the external anal sphincter during straining to defecate (simulated defecation) in the EMG-BF group during the baseline was 10.03, and in the control group was 10.97.

**Figure 2:**
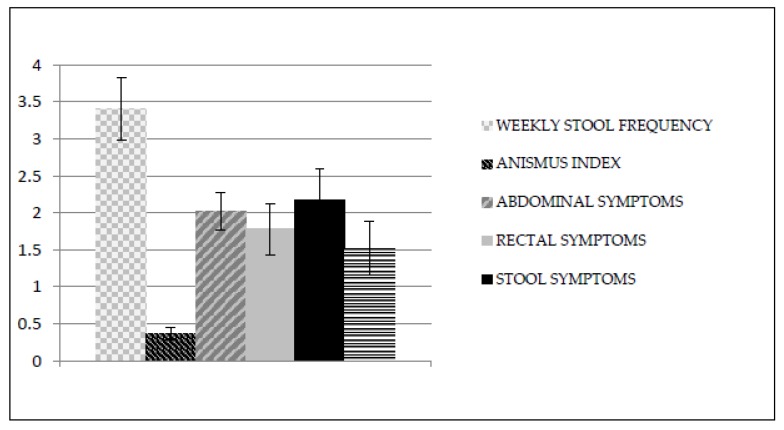
Mean values in all dependent measures after treatment in the EMG-biofeedback group.

**Figure 3:**
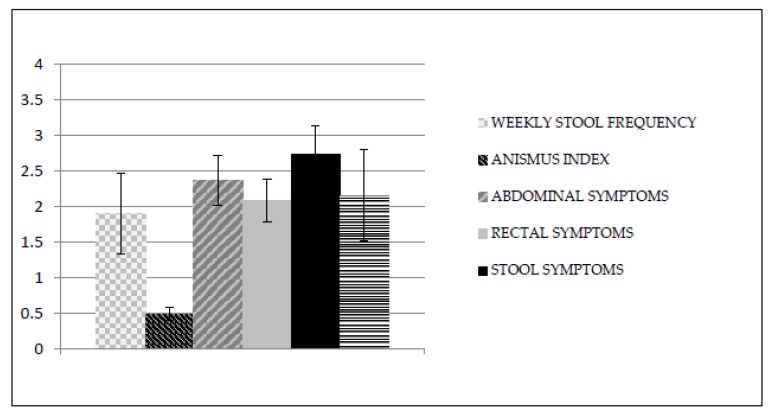
Mean values in all dependent measures after treatment in the control group.

After treatment, MANOVA revealed significant differences between the groups (Wilks’s λ = 0.29; F_6,13_ = 5.19; *p* < 0.01). These differences were statistically significant in all dependent measures (Table 2): anismus index (F_1,18_ = 9.89; *p* < 0.01), weekly stool frequency (F_1,18_ = 19.70; *p* < 0.01), abdominal symptoms (F_1,18_ = 5.63; *p* < 0.05), rectal symptoms (F_1,18_ = 4.56; *p* < 0.05), stool symptoms (F_1,18_ = 8.89; *p* < 0.01), and overall PAC-QOL scale (F_1,18_ = 6.92; *p* < 0.05). Moreover, the mean EMG-activity (µV) of the external anal sphincter during straining to defecate in the EMG-BF group after treatment was 4.99, while in the control group it remained virtually unchanged (10.16 uV). Graphic representation of the mean values in all dependent measures after treatment in the EMG-biofeedback group and in the control group can be seen in Figure 2 and Figure 3, respectively.

Finally, the differences in the scores obtained in all the variables before and after the treatment in each of the groups were analyzed. MANOVA for the control group revealed no significant differences (Wilks’s λ = 0.30; F_6,4_ = 1.58; *p* = 0.34). Univariate analysis showed no differences in any of the measured dependent variables: anismus index (F_1,9_ = 0.28; *p* = 0.61), weekly stool frequency (F_1,9_ = 3.86; *p* = 0.08), abdominal symptoms (F_1,9_ = 0.64; *p* = 0.44), rectal symptoms (F_1,9_ = 2.04; *p* = 0.19), stool symptoms (F_1,9_ = 0.01; *p* = 0.95), and overall PAC-QOL scale (F_1,9_ = 3.27; *p* = 0.10). By contrast, MANOVA for the EMG-BF group revealed statistically significant differences (Wilks’s λ = 0.02; F_6,4_ = 40.81; *p* < 0.01). These differences were significant in all the dependent variables: anismus index (F_1,9_ = 74.22; *p* < 0.01), weekly stool frequency (F_1,9_ = 73.50; *p* < 0.01), abdominal symptoms (F_1,9_ = 30.37; *p* < 0.01), rectal symptoms (F_1,9_ = 13.90; *p* < 0.01), stool symptoms (F_1,9_ = 20.84; *p* < 0.01), and overall PAC-QOL scale (F_1,9_ = 65.61; *p* < 0.01).

## 4. Discussion

This randomized controlled trial showed the efficacy of EMG-BF for the management of chronic constipation due to dyssynergic defecation in elderly women. Significant improvements in anismus index; weekly stool frequency; and abdominal, rectal, and stool symptoms were achieved in the participants who received behavioral treatment compared to controls who received counselling and information about bowel functioning and the behavioural and psychophysiological mechanisms involved in the defecation process. These results are consistent with those of previous research that informed the clinical gains following behavioral treatment with biofeedback in dyssynergic defecation patients [10,11,12,13,14], particularly with the results obtained by our research group in elderly women [19,31]. 

Furthermore, to our knowledge, this is the first controlled study aimed at evaluating the possible impact of biofeedback therapy on the improvement of quality of life in elderly women with dyssynergic defecation. The results obtained confirm the significant benefits on the health-related quality of life of the women participants achieved after behavioral treatment. This finding has salient implications for the development of specific treatment programs for the management of dyssynergic defecation in elderly women.

This study presents some limitations. Probably, the most important are the small sample size, the relatively short time of the follow-up, and the absence of measures of psychological distress. This issue is very important, because defecation disorders are often associated with psychopathological dysfunctions [32] For this reason, studies aimed at analyzing the psychological state, particularly the prevalence of dysfunctions such as anxiety and depression in elderly women with dyssynergic defecation, must be carried out in the future. Furthermore, given that behavioral treatment through biofeedback produces improvements in both the symptoms of constipation and in the health-related quality of life, future research in this field should determine the effects that these improvements could have on the psychological well-being of these patients.

## 5. Conclusions

The results of this study provide evidence of the benefits of behavioral treatment through EMG-biofeedback, both in terms of clinical symptoms and health-related quality of life in elderly women with dyssynergic defecation.

## Figures and Tables

**Table 1:** Anismus index, weekly stool frequency, clinical symptoms, and overall quality of life (QOL) measure before and after treatment in the electromyographic biofeedback (EMG-biofeedback) group and control group.

Dependent Variable	*EMG-Biofeedback*			*Control Group*		
	*Baseline*	*After Therapy*	*p*	*Baseline*	*After Therapy*	*p*
*AI*	0.58 (±0.14)	0.37 (±0.08)	<0.01	0.50 (±0.11)	0.49 (±0.09)	0.61
*WSF*	1.80 (±0.42)	3.40 (±0.42)	<0.01	1.60 (±0.52)	1.90 (±0.57)	0.08
*ABD-SYM*	2.47 (±0.40)	2.02 (±0.25)	<0.01	2.32 (±0.31)	2.37 (±0.35)	0.44
*REC-SYM*	2.08 (±0.26)	1.78 (±0.35)	<0.01	2.20 (±0.39)	2.09 (±0.30)	0.19
*STOOL-SYM*	2.94 (±0.36)	2.17 (±0.43)	<0.01	2.74 (±0.34)	2.73 (±0.41)	0.95
*Overall PAC-QOL scale*	2.41 (±0.57)	1.52 (±0.36)	<0.01	2.25 (±0.59)	2.16 (±0.65)	0.10

*Note:* data are expressed as mean (±standard deviation, SD). AI = anismus index; WSF = weekly stool frequency; ABD-SYM = abdominal symptoms; REC-SYM = rectal symptoms; STOOL-SYM = stool symptoms; and PAC-QOL = patient assessment of constipation-quality of life.

**Table 2:** Analysis of the differences between EMG-biofeedback group and control group in the baseline and after therapy for each dependent variable.

*Dependent Variable*	*Baseline*		*After Therapy*	
	***F***	***p***	***F***	***p***
*AI*	2.43	0.14	9.89	<0.01
*WSF*	0.90	0.35	19.70	<0.01
*ABD-SYM*	0.79	0.38	5.63	<0.05
*REC-SYM*	0.60	0.45	4.56	<0.05
*STOOL-SYM*	1.55	0.23	8.89	<0.01
*Overall PAC-QOL scale*	0.38	0.54	6.92	<0.05
